# Participation of Children and Youth with and Without Cerebral Palsy Across Settings: An Exploratory Study

**DOI:** 10.3390/children12060707

**Published:** 2025-05-29

**Authors:** Teresa Pierce, Alyssa LaForme Fiss

**Affiliations:** 1 Department of Physical Therapy, Philadelphia College of Osteopathic Medicine, Georgia Campus, Suwanee, GA 30024, USA; 2 School of Physical Therapy, Texas Woman’s University, Denton, TX 76204, USA

**Keywords:** participation, environment, children, cerebral palsy

## Abstract

**Background/Objective:** Children with disabilities experience greater participation restrictions in life activities than children without disabilities. This study aimed to explore differences in participation of children/youth with and without cerebral palsy (CP) across home, school, and community settings, and examine participation of children with CP across gross motor function levels, age groups, gender, and income levels. **Methods:** This was a cross-sectional survey study of parents of children with CP (*n* = 20) and without CP (*n* = 20) over a three-month period to investigate the frequency of participation/level of involvement across settings. Participation was assessed using the Participation and Environment Measure for Children and Youth© (PEM-CY), a parent-report measure for children and youth, ages 5 to 17 that explores communication in home, school, and community environments. **Results:** Significantly greater frequency of participation at home and in the community was found in children without CP. Participation at school was not significantly different between the groups and there were no differences in level of involvement, gender, or income. In children with CP, motor function impacted participation in home and school, and age significantly influenced school participation. **Conclusions:** Participation in life activities is important for overall child development, health, and well-being; however, children with CP participate less than their typically developing peers. The ability to collect and analyze the frequency of participation and level of involvement across home, school, and community environments using one outcome measure provides valuable information for programming, intervention planning, and potential environment modifications that could improve participation in children with CP.

## 1. Introduction

Meaningful participation and involvement in everyday activities and routines can have a positive influence on children’s well-being, development, and optimal health [1]. The World Health Organization (WHO) defines participation as “involvement in life activities” [2]. Children with disabilities experience greater participation restrictions in life activities than children without disabilities [3,4,5,6]. The most common cause of chronic, severe childhood disability is cerebral palsy (CP), a non-progressive, neurological condition with a prevalence of 2.11 per 1000 live births, internationally [7]. It is well documented in the literature that children and youth with CP participate less than their typically developing peers [8,9,10,11].

Historically, the participation of children with CP has been assessed using outcome measures that focused on functional disabilities and limitations [12]. However, the WHO moved away from the disablement model of health in 2001 to promote a biopsycho-social approach called the International Classification of Function, Disability, and Health, or the ICF model. This model emphasizes meaningful activities and participation, along with environmental and personal factors [13]. In 2007, the ICF model was revised to address pediatric health conditions and, as a result, the assessment of children with disabilities underwent a significant paradigm shift, focusing more on evaluating involvement in school and leisure activities [5,14]. In their scoping review on the effect of environment on participation, Anaby et al. [15] noted that the environment was the “key factor influencing participation” in children and youth with disabilities and environmental factors significantly impacted participation [16]. The WHO defines environment as “the physical, social, and attitudinal environment in which people live and conduct their lives” [2].

Research examining child participation in the context of the environment is limited [17]; however, several studies have investigated the impact of environmental factors on participation in school-aged children/youth with CP. A multi-center study of children with CP associated participation with social, physical, and attitudinal environment, and found that environment explained 14% to 52% of the variation in participation [18]. Mitchell et al. [19] explored environmental characteristics of ambulatory children/youth with CP and found that participation in the home and community was associated with increased physical activity, but the model was somewhat weak (*R*^2^ = 0.32). A Serbian study explored the home and community participation of children with CP and found that children with CP participated less than children with typical development [20].

Physical characteristics that impact the participation of children with CP, such as gross motor ability, have been examined. Individuals with CP have varying degrees of movement limitations. Gross motor ability is classified from levels I (mild impairment) to V (severe impairment) using the Gross Motor Function Classification System (GMFCS) [21]. Gross motor ability, or GMFCS level, has been correlated with participation—children with smaller GMFCS levels were reported to participate more often and with greater intensity than children with larger GMFCS levels [22,23].

Other social characteristics affecting participation in children with CP, such as age and gender, have also been investigated with inconsistent results. A few studies found that younger age was associated with greater participation [22,24]. In contrast, authors of a multi-site study of children with CP in the U.S. and Canada (*n* = 694) found that differences in participation were significantly correlated (*p* < 0.001, *η^2^* = 0.20) with motor function but not age and gender [25]. A retrospective Australian study of children with CP reported that girls participated in more activities than boys [10].

In addition, there is limited evidence correlating family income with the participation of children with CP. Law et al. [6] noted that participation of children with disabilities was “less diverse” (meaning fewer variety of activities) in low-income families. Palisano et al. [22] included income as part of a “family characteristic” variable in an analysis of determinants of participation in a study with 288 children with CP, ages 6 to 12 years. Results indicated that greater family activity orientation was associated with greater child participation (*β* = 0.27, *p* ≤ 0.05) [22].

Participation in home, school, and community is a fundamental right for all children and can provide meaningful life experiences for individuals with CP [14]. The ability to objectively measure participation across multiple environments is not only sensible, but provides extrinsic knowledge about frequency and level of involvement in daily life activities, which could provide valuable knowledge of child engagement and enhance therapeutic intervention across settings [24]. Therefore, the aims of this exploratory study were three-fold: (1) to explore differences in participation of children/youth with and without CP across home, school, and community settings, (2) to examine the participation of children with CP across gross motor function levels, age groups, gender, and income levels in those settings, and (3) to determine the feasibility of larger, future studies investigating participation across settings.

## 2. Materials and Methods

A cross-sectional design was used to collect parent-reported survey data in March, April, and May 2022. This exploratory study was approved by the Institutional Review Board (IRB) of the Philadelphia College of Osteopathic Medicine (PCOM). The study was deemed exempt as all responses were collected anonymously via an electronic survey and stored in a secure REDCap^®^ account at PCOM.

A convenience sample of parents of children with a diagnosis of CP was recruited from nine local outpatient pediatric therapy clinics in a large suburban area. A second convenience sample of parents of local children without CP who were developing typically was recruited for comparison. Inclusion criteria for the study were specified for the two groups. Parents of children with CP had to (1) be over 18 years of age, (2) be a parent of a child between 5 and 17 years of age with a diagnosis of CP, and (3) be able to read English. Parents of children without CP had to (1) be over 18 years of age, (2) be a parent of a child between ages 5 and 17 years of age who is developing typically with no medical diagnosis, and (3) be able to read English. Parents of children from both groups were excluded if the child had undergone surgery in the last six months. A screening form with inclusion/exclusion criteria YES/NO questions was used to recruit eligible participants. Investigators recruited 20 parents of children with CP and 20 parents of children without CP, with children of ages 5 to 17.

Once recruited, participants received a hyperlink to begin the electronic survey. The first display was a Participant Information form describing the study. At the end of the form, participants were directed to a checkbox with instructions to click on the box to give consent to participate in the study. Under the consent box was an email link to the principal researcher in case the participant had questions about the study or study process. Once the participant consented by checking the consent box, a link to the survey was visible that also included contact information for the principal researcher and a PCOM IRB representative.

**Figure 1:**
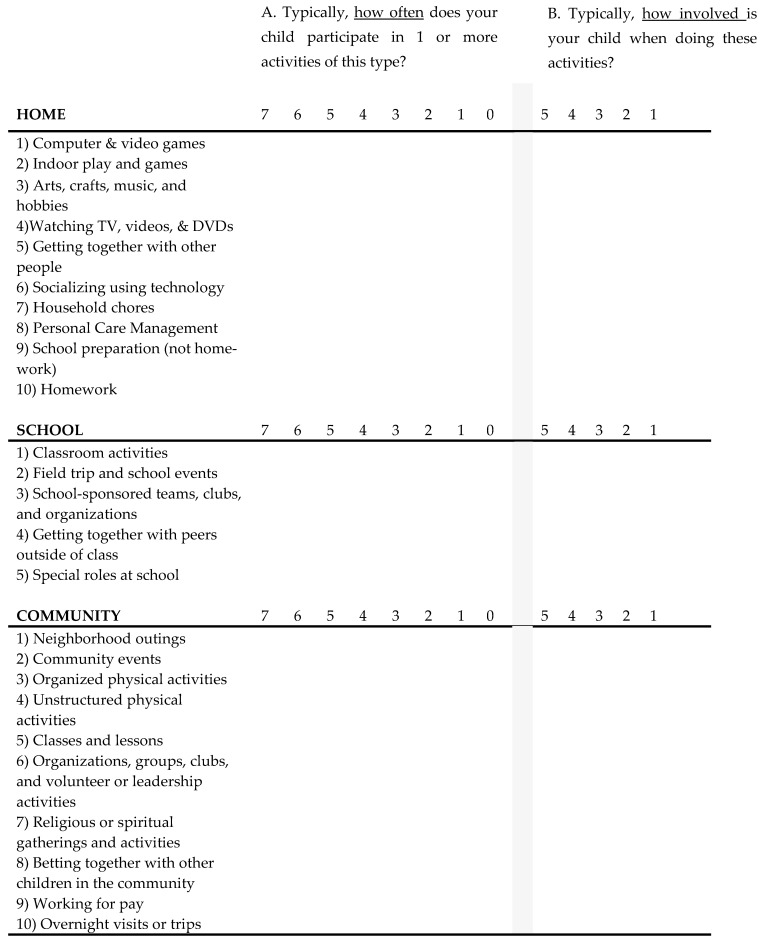
Participation and Environment Measure for Children and Youth©. Question A: 7 = daily, 6 = a few times a week, 5 = once a week, 4 = a few times a month, 3 = once in the last 4 months, 2 = a few times in the last 4 months, 1 = once in the last 4 months, 0 = never. Question B: 5 = very involved, 4 = between very involved and somewhat involved, 3 = somewhat involved, 2 = between somewhat involved and minimally involved, 1 = minimally involved.

Survey data related to child participation were collected using the Participation and Environment Measure for Children and Youth© (PEM-CY), a parent-report measure for children and youth, ages 5 to 17. [Henceforth, the term *children* will be used to describe children and youth, ages 5 to 17.] The PEM-CY is the first measure to evaluate participation in important activities across three settings, or environments—home, school, and community. This measure assesses the frequency of participation and level of involvement, including perceived barriers and supports, as well as whether parents would like to see their child’s participation change, and, if so, how they would like it to change [26]. The PEM-CY uses a Likert scale to measure participation frequency and level of involvement. The home and community sections have 10 items each, and the school section has 5 items. Parents are asked to identify how frequently over the past four months the child participated in the listed activities (daily to never = eight choices) and how involved their child is when participating (very involved to minimally involved = five choices). (See Figure 1 for PEM-CY questions used in this study.)

Psychometric properties of the PEM-CY indicate adequate reliability and validity. Test–retest reliability was moderate to good for all participation and environment scores (ICC = 0.58–0.95) and across items within home, school, and community sections (ICC = 0.68–0.96). There were large and significant differences between groups with and without disabilities, demonstrating discriminant validity [26]. Internal consistency was also moderate to good for participation frequency (*α* = 0.59–0.70) and participation involvement (*α* = 0.72–0.83). Concurrent validity of the PEM-CY was established, as noted by moderate to strong associations between the Craig Hospital Inventory of Environmental Factors for Children-Parent Version and PEM-CY scores (*r* = −0.43 to 0.64) [27].

An anonymous electronic survey was created in REDCap^®^ and included demographic questions in addition to the PEM-CY queries. Participants were asked their date of birth, gender, race, household income, child date of birth, child gender, and if their child had CP. If they answered “yes”, to indicate their child had CP, a dialogue box opened that asked the child’s age range—less than 6, 6 to 11, or 12 to 17 years. [These age groups were established to coincide with the CP GMFCS rating system.] Participants then determined their child’s GMFCS level by choosing one response from a series of statements describing their child’s mobility, available from the *CanChild Foundation* [28]. This self-rating system for GMFCS-level classification showed a high correlation (97.8%) between parent and therapist responses [29].

The survey was piloted with two parents and two therapists who provided feedback on the demographic questions, which were modified based on pilot suggestions. A recruitment packet was delivered to each clinical partner. The packet included a Participant/Recruitment Log, Participant Screening forms, and Participant Information forms. The principal investigator provided clinical partners with written and verbal instructions for recruitment and information to share with participants. Clinical partners were contacted approximately every 7–10 days by phone or email to follow up on recruitment and field any questions. The principal investigator accessed REDCap^®^ two to three times a week to view data collection and check participant response numbers. The collection of survey data continued for three months. Forty-eight parents were recruited for this study and forty returned the survey (62.5%), which is considered an excellent response [30].

Data analysis was performed using the IBM SPSS 28 program. For this exploratory study, the dependent variable was PEM-CY scores for frequency of participation and level of involvement. Internal consistency was measured for all items—frequency of participation in home, school, and community activities, along with level of involvement in home, school, and community activities. Cronbach’s α for the six subscales was α ≥ 0.701 for all items, indicating adequate internal consistency.

Chi-square analysis was performed to look for a significant association between the nominal variables, gender, and diagnosis of CP. Continuous data were inspected for normality, outliers, and missing data. Non-normal distributions were noted in home frequency, school involvement, and community involvement, found by viewing histograms and calculation of several significant Kolmogorov–Smirnov and Shapiro–Wilk values, although skewness and kurtosis were acceptable (<±2) [31]. Three cases (7.5%) from the sample were missing large amounts of data and were removed from the data set, following guidelines for managing data missing at random (MAR) [32]. One value was missing from home involvement total scores and Winsorization was used to transform that score to the next closest value [33]. Four outliers were found—two in frequency at school and two in level of involvement at school—but were left in the data set to allow for a complete analysis of the survey responses.

Due to normality violations and the small sample size, non-parametricbivariate analyses were performed. Mann–Whitney U tests were used to explore differences between children with CP and children without CP in frequency of participation and level of involvement in all environments. Mann–Whitney U tests were also run to investigate the frequency of participation in children with CP across GMFCS levels and gender. Kruskal–Wallis H tests were used to examine differences in frequency of participation in children with CP by age group and family income, including post hoc pairwise comparisons with a Bonferroni correction. Effect sizes were computed for all significant differences. The values of the effect sizes can be interpreted according to the following guidelines: Cohen’s *d*, *d* = 0.20 indicates a small effect, *d* = 0.50 indicates a medium effect, and *d* = 0.80 indicates a large effect size. For eta-squared, *η*^2^ = 0.01 indicates a small effect, *η*^2^ = 0.06 indicates a medium effect, and *η*^2^ = 0.14 indicates a large effect size [34].

## 3. Results

A total of 40 parents participated in the exploratory survey study, but only 37 surveys were included in data analysis due to a significant number of omitted responses on 3 of the surveys. Since the number of surveys missing data was small and missing at random, authors opted not to use only complete cases [32]. There were 18 parents of children with CP and 19 parents of children without CP that completed the entire survey. The Chi-square test of independence showed no significant association between gender and the diagnosis of CP, *Χ*^2^ (1, *N* = 37) = 0.83, *p* = 0.362. Descriptive statistics are listed for participant characteristics in Table 1, and participation scores between the groups are summarized in Table 2.

Home participation was significantly greater in children without CP (Median or Mdn = 6.20), *U* = 77.00, *z* = −2.86, *p* = 0004, *r* = 0.47, when compared to children with CP (Mdn = 4.10). G*Power analysis of home participation between the groups revealed a power (1–β err prob) of 0.27 for a two-tailed test with 18 participants in the group with CP and 19 in the group without CP with the following parameters: effect size 0.47 in the home environment and an alpha (α err prob) of 0.05.

There were also significant differences in the frequency of participation in community activities with children without CP (Mdn = 3.60), scoring greater than children with CP (Mdn = 2.20), *U* = 54.50, *z* = −3.54, *p* < 0.001, *r* = 0.58. G*Power analysis of community participation between the groups revealed a power (1–β err prob) of 0.39 for a two-tailed test with 18 participants in the group with CP and 19 in the group without CP with the following parameters: effect size 0.58 in a community environment and an alpha (α err prob) of 0.05.

The frequency of school participation was not significantly different in children with CP (Mdn = 3.40) and children without CP ((Mdn = 4.20). No significant differences were found in the level of involvement between the groups with and without CP (see Table 3).

Comparison of frequency of participation at home across GMFCS levels showed significant differences between levels I, II, and III (Mdn = 5.80) and levels IV and V (Mdn = 4.00), *U* = 11.50, *z* = −2.46, *p* = 0.014, *r* = 0.40, with greater participation of individuals with GMFCS levels I, II, and III. G*Power analysis of home participation between GMFCS groups revealed a power (1–β err prob) of 0.12 for a two-tailed test with 11 participants in the GMFCS I-III group and 7 in the group with GMFCS IV-V with the following parameters: effect size 0.40 in the home environment and an alpha (α err prob) of 0.05.

Individuals with GMFCS levels I, II, and III showed a greater frequency of school participation (Mdn = 4.00) than levels IV and V (Mdn = 1.60), *U* = 13.50, *z* = −2.27, *p* = 0.023, *r* = 0.37. G*Power analysis of school participation between GMFCS groups revealed a power (1–β err prob) of 0.11 for a two-tailed test with 11 participants in the GMFCS I–III group and 7 in the group with GMFCS IV-V with the following parameters: effect size 0.37 in the school environment and an alpha (α err prob) of 0.05.

No significant differences were noted in the frequency of community participation based on GMFCS levels. Also, no significant differences were found in the frequency of participation in any of the environments based on gender (Table 4).

In children with CP, the frequency of participation at home was not impacted by age, *H*(2) = 4.32, *p* = 0.115, nor was participation in the community, *H*(2) = 3.58, *p* = 0.167). However, the frequency of participation at school for children with CP was significantly impacted by age, *H*(2) = 10.44, *p* = 0.005. Pairwise comparisons with adjusted p-values showed significant differences in school participation between children less than 6 years old and children 12 to 17 years old (*p* = 0.013, *η*^2^ = 0.11) and between children less than 6 years old and children 6 to 11 years old (*p* = 0.002, *η*^2^ = 0.07), with younger children participating less at school (Table 5). G*Power analysis of school participation between youngest and oldest groups revealed a power (1–β err prob) of 0.51 for a two-tailed test with 18 participants with the following parameters: effect size 0.59 in the school environment and an alpha (α err prob) of 0.05. G*Power analysis of school participation between youngest and middle age groups revealed a power (1–β err prob) of 0.71 for a two-tailed test with 18 participants with the following parameters: effect size 0.73 in the school environment and an alpha (α err prob) of 0.05.

No significant differences were noted between 12- and 17-year-olds and 6- and 11-year-olds (*p* = 0.748, *r* = 0.08). Additionally, no significant differences were found in participation of children with CP by household income in any setting (Table 6).

## 4. Discussion

Environment is a key determinant of child participation [15]. Few studies have explored the participation of children with CP in different settings as assessment tools rarely include data collection across multiple environments. This exploratory study used the PEM-CY to investigate the participation of children/youth with and without CP across home, school, and community settings. Using this tool to assess participation across settings demonstrates the novelty of this study as the PEM-CY is the first measure to assess participation in three environments [26]. Additionally, only a small number of studies have used the PEM-CY to examine the participation of children with cerebral palsy [9,19,20,35,36,37]. Participation of children with CP was also explored across gross motor function levels, age groups, gender, and income levels. The validity of this exploratory study was strengthened by the results of the Chi-square test of categorical data which were consistent with the results of a large birth prevalence study that also reported no significant association of gender with a diagnosis of CP [38].

Data for this study were compiled from responses to survey questions on the PEM-CY that addressed the frequency of participation and level of involvement. The frequency of participation in children without CP in home and community environments was significantly greater than participation in children with CP (*p* = 0.004, *d* = 0.47 and *p* < 0.001, *d* = 0.58, respectively), as demonstrated in many earlier studies [3,4,5,6,39], with medium effect sizes. However, a unique finding of this study was that school participation between children with and without CP was not significantly different. Perhaps this is an indication that education systems are reducing barriers, supporting accommodations, improving accessibility, and moving towards equal access—all positive implications.

Another interesting finding of this study was that the level of involvement across all environments was not significantly different for children with and without CP, contrary to involvement findings from earlier research by Milićević and Nedović [20], yet, similar to results from Di Lieto, et al. [35], who found no differences in level of involvement. It is difficult to discern the validity of these findings, as the survey tool may not be sensitive enough to detect meaningful differences in levels of involvement in this population.

Significant differences in participation were found based on the gross motor function of children with CP. Although effect sizes were small to medium, ambulatory children with CP (GMFCS I–III) demonstrated greater frequency of participation in home and school environments (*p* = 0.014, *d* = 0.40 and *p* = 0.023, *d* = 0.37, respectively), which aligns with results of earlier studies [10,22]. However, there were no significant differences in community participation between ambulatory and non-ambulatory children. This positive finding of similar frequency of participation in the community could possibly be due to advances in access and technology of mobility devices, improved community accessibility, and increased opportunities for non-ambulatory children to participate in community activities.

The impact of gender and age on the frequency of participation in children with CP was varied. In this sample, there were no significant differences in participation between males and females with CP, aligning with results from the *On*-*Track* study [25]. The frequency of participation was not impacted by age in home and community environments; however, school participation was significantly different between age groups. Differences were noted between the younger and older groups (*p* = 0.013, *η*^2^ = 0.11) and between the younger and middle groups (*p* = 0.002, *η*^2^ = 0.07), with younger children participating less, with a medium effect, despite the small sample size. These results do not align with previous studies that found the participation of children with CP decreased with age [22,24]; however, the youngest group in the present study was less than six and may not yet have been in many school activities.

Results showed that participation was not affected by household income, contrary to earlier research indicating that reduced income was associated with less frequent participation [6,22]. The lack of a significant difference could be due to the socio-economic demographic of our sample—a large metropolitan suburb with a high-quality educational system, disability services, and access to community resources and public facilities. Additionally, the lack of variability in household income could have impacted results as the group with CP was evenly split between the three income categories, with two-thirds of the families with CP categorized as middle-class and upper-middle class [40].

Several strengths of the study were noted. First, this exploratory study investigated child participation across environments using a single outcome measure, which is an innovative and efficient research approach [17]. The PEM-CY reflects “the interactive relationship between health conditions and contextual factors” reflected in the ICF model [13,14]. Second, the frequency of participation in children with CP was explored across several variables—age, gender, GMFCS level, and household income. Statistical analysis demonstrated significant differences in multiple comparisons, justifying the need to pursue further analysis to thoroughly examine additional factors impacting the participation of children with CP. Lastly, using REDCap^®^, a secure web platform for survey data collection and storage, allowed participants a convenient, anonymous method for study participation.

Potential weaknesses of this study included a small number of participants, a single geographical area, and variability within the sample. The small sample size limited study power and grouping options [30]. Maximum power achieved with Mann–Whitney analysis comparing participation of children in this study with (*n* = 18) and without (*n* = 19) CP was (1–β err prob) 39%. Larger future studies are needed as an a priori estimate from G*Power indicated that two groups with 67 participants each are required to achieve 80% power on a two-tailed test with an effect size of 0.50 and an alpha of 0.05.

Convenience sampling was used for participant recruitment from outpatient pediatric clinics in suburban regions of one state, which may not be representative of the population with CP. Due to this possible sampling error, results may not be generalizable. In addition, participant responses were collected via an electronic, anonymous survey, which increases the possibility that respondents provided “hasty or less thoughtful” responses [26]. As with many studies related to children with CP, considerable variation existed when results were compared with previous studies. This variation is likely due, in part, to the heterogeneity of characteristics in children with CP [7] and the use of different outcome measures across studies. Differences in frequency of participation were found among GMFCS groups and age groups. However, GMFCS groups may not have adequately reflected all gross motor levels since the children with CP were grouped into only two categories—ambulatory and non-ambulatory. Three age groups were established for children with CP (<6, 6 to 11, and 12 to 17), which yielded a much smaller age range in the younger group since the PEM-CY is only validated for children and youth 5 to 17 years old.

The benefit of child participation as a key indicator of health and development is well established [1]. Participation is a complex construct that is influenced by a variety of child, family, and environmental factors [3,8,10]. Research has shown that activity and participation decline with age in children with CP [5]; therefore, the ability to assess and monitor participation in different environments could help families and providers identify strategies for improving participation. Optimizing a child’s ability to participate in home, school, and community activities is an important outcome of pediatric rehabilitation programs [22] and positively influences quality of life [11,35,41].

In future studies, large and equal samples are needed to broaden these results with parametric analyses. Additionally, longitudinal studies using the PEM-CY could provide valuable parent-reported data about change over time. Investigating factors that impact participation across home, school, and community environments could supply helpful information for making policy decisions and improving access. Further research is also needed to evaluate the influence of child/family characteristics on all aspects of participation.

## 5. Conclusions

The results of this exploratory study corroborate previous findings that children with CP participate less frequently than children without CP in home and community environments, but add to the body of knowledge related to participation research in that school participation was not significantly different in this sample. Personal factors, such as the age and GMFCS level of children with CP, significantly impacted participation in some settings. Findings from this exploratory study support the feasibility of using a single measure, like the PEM-CY, to conduct larger studies investigating participation across multiple settings. Participation data from home, school, and community environments could help clinicians identify patterns and develop intervention plans that focus on maximizing child participation in all settings.

## Figures and Tables

**Table 1:** Demographics and descriptive statistics of study participants and their children.

Participants	*n*	Percentage	Mean (SD)	Range
Parent/Caregiver: AGE (years) Sex Male Female Race White Black/African American Other Household Income <$52,200 $52,201–$156,000 >$156,000	37 * 9 28 29 3 5 7 16 14	24.3% 75.6% 78.3% 8.1% 13.5% 18.9% 43.2% 37.8%	42.51 (9.11)	24–60
Children without CP: AGE (years) <6 6 to 11 12 to 17 Sex Male Female Household Income <$52,200 $52,201–$156,00 >$156,000	19 3 11 5 11 8 1 10 8	15.7% 57.9% 26.3% 57.9% 42.1% 5.2% 52.6% 42.1%	8.61 (4.08)	5–17
Children with CP: AGE (years) <6 6 to 11 12 to 17 Sex Male Female Household Income <$52,200 $52,201–$156,000 >$156,000 GMFCS Level: I II III IV V	18 5 8 5 13 5 6 6 6 1 5 5 4 3	27.7% 44.4% 27.7% 72.2% 27.7% 33.3% 33.3% 33.3% 5.5% 27.7% 27.7% 22.2% 16.6%	8.84 (3.45)	5–16

Key: * denotes the actual number of cases analyzed for this study as three participants were removed due to significant missing survey responses.

**Table 2:** Summary of environmental participation scores by group.

PEM-CY Scores	Children Without CP *n* = 19		Children with CP *n* = 18	
**Home** Frequency of Participation Level of Involvement	**Range**	**Mean (SD)**	**Range**	**Mean (SD)**
	4.80–7.00 3.30–5.00	6.10 (0.63) 4.23 (0.60)	3.10–6.80 2.60–5.00	4.82 (1.29) 3.80 (0.77)
**School** Frequency of Participation Level of Involvement	1.00–6.40 1.50–5.00	3.91 (0.27) 4.23 (0.95)	0.00–7.00 1.50–5.00	3.24 (2.10) 3/72 (1.35)
**Community** Frequency of Participation Level of Involvement	2.30–5.50 1.00–5.00	3.62 (0.89) 3.94 (1.22)	0.60–5.70 1.33–5.00	2.39 (1.12) 3.47 (1.01)

Key: PEM-CY = Participation and Environment Measure-Children and Youth; CP = cerebral palsy.

**Table 3:** Non-parametric tests for participation frequency and level of involvement between groups.

A. Frequency of Participation							
Home							
	n	MEAN RANKS	Median	U	z	p	d
No CP	19	23.95	6.20	77.00	−2.86	0.004 *	0.47
CP	18	13.78	4.10				
School							
	n	MEAN RANKS	Median	U	Z	p	d
No CP	19	21.50	4.20	123.50	−1.45	0.148	-
CP	18	16.36	3.40				
Community							
	n	MEAN RANKS	Median	U	Z	p	d
No CP	19	25.13	3.60	54.50	−3.54	<0.001 *	0.58
CP	18	12.53	2.20				
B. Level of Involvement							
Home							
	n	MEAN RANKS	Median	U	z	p	
No CP	19	22.13	4.30	115.50	−1.81	0.070	
CP	18	15.69	3.83				
School							
	n	MEAN RANKS	Median	U	Z	p	
No CP	19	20.26	4.60	147.00	−0.746	0.456	
CP	18	17.67	4.23				
Community							
	n	MEAN RANKS	Median	U	Z	p	
No CP	19	21.79	4.14	118.00	−1.62	0.106	
CP	18	16.06	3.31				

Key: * denotes significance; MEAN RANKS refer to the average of the ranking, or position, of scores in a data set [27].

**Table 4:** Analysis of frequency of participation in home, school, and community across GMFCS levels and gender.

A. Frequency of Participation							
Home							
GMFCS level	n	MEAN RANKS	Median	U	z	p	d
I to III	11	11.95	5.80	11.50	−2.46	0.014 *	0.40
IV and V	7	5.64	4.00				
School							
	n	MEAN RANKS	Median	U	Z	p	d
I to III	11	11.77	4.00	13.50	−2.27	0.023 *	0.37
IV and V	7	5.93	1.60				
Community							
	n	MEAN RANKS	Median	U	Z	p	d
I to III	11	10.73	2.40	25.00	−1.23	0.221	-
IV and V	7	7.57	1.90				
Home							
GENDER	n	MEAN RANKS	Median	U	z		p
Male	13	8.65	4.10	21.50	−1.09		0.275
Female	5	11.70	5.80				
School							
	n	MEAN RANKS	Median	U	z		p
Male	13	9.69	3.20	30.00	−0.247		0.805
Female	5	9.00	3.60				
Community							
	n	MEAN RANKS	Median	U	z		p
Male	13	9.50	2.10	32.50	0.00		1.000
Female	5	9.50	2.40				

Key: * denotes significance.

**Table 5:** Analyses of frequency of participation in home, school, and community across age groups in children with CP.

A. Frequency of Participation						
				Home		
AGE GROUP (years)	n	MEAN RANKS	Median	H	df	p
<6	5	7.70	4.00	4.32	2	0.115
6 to 11	8	12.38	6.10			
12 to 17	5	7.70	4.00			
				School		
	n	MEAN RANKS	Median	H	df	p
<6	5	3.00	1.40	10.44	2	0.005 *
6 to 11	8	12.38	4.40			
12 to 17	5	11.40	3.80			
				Community		
	n	MEAN RANKS	Median	H	df	p
<6	5	9.40	2.40	3.58	2	0.167
6 to 11	8	11.75	2.65			
12 to 17	5	6.00	1.50			
Pairwise Comparisons of Age Groups and School Participation						
Age Groups			Test Statistic (H)		p	r
Less than 6 years old & between 12 to 17 years old			−2.50		0.013 *	−0.59
Less than 6 years old & between 6 to 11 years old			−3.10		0.002 *	−0.73
Between 12 to 17 years old & between 6 to 11 years old			0.32		0.748	0.08

Key: * denotes significance.

**Table 6:** Analyses of frequency of participation in home, school, and community across household income in children with CP.

A. Frequency of Participation						
				Home		
Household INCOME	n	MEAN RANKS	Median	H	df	p
<$52,200	6	18.57	5.80	2.81	2	0.245
$52,201 to $156,600	6	22.19	6.20			
>$156,600	6	15.57	5.40			
				School		
	n	MEAN RANKS	Median	H	df	p
<$52,200	6	21.93	4.80	1.39	2	0.500
$52,201 to $156,600	6	16.72	3.30			
>$156,600	6	20.14	4.00			
				Community		
	n	MEAN RANKS	Median	H	df	p
<$52,200	6	19.29	3.10	0.59	2	0.745
$52,201 to $156,600	6	20.34	2.95			
>$156,600	6	17.32	2.65			

## Data Availability

Supplemental data are available from the corresponding author upon request.

## Abbreviations

The following abbreviations are used in this manuscript:

CP	Cerebral Palsy
GMFCS	Gross Motor Function Classification System
ICF	International Classification of Function, Disability, and Health
IRB	Institutional Review Board
PEM-CY	Participation and Environment Measure for Children and Youth
US	United States
WHO	World Health Organization
